# Growth Differentiation Factor-15 as a Potent Predictor of Long-Term Mortality among Subjects with Osteoarthritis

**DOI:** 10.3390/jcm9103107

**Published:** 2020-09-26

**Authors:** Natalie Arnold, Martin Rehm, Gisela Büchele, Raphael Simon Peter, Rolf Erwin Brenner, Klaus-Peter Günther, Hermann Brenner, Wolfgang Koenig, Dietrich Rothenbacher

**Affiliations:** 1 Department of General and Interventional Cardiology, University Heart Centre Hamburg, 20251 Hamburg, Germany; n.arnold@uke.de; 2 German Center for Cardiovascular Research (DZHK), partner site Hamburg/Kiel/Luebeck, 20251 Hamburg, Germany; 3 Institute of Epidemiology and Medical Biometry, Ulm University, 89081 Ulm, Germany; martin.rehm@uni-ulm.de (M.R.); Gisela.buechele@uni-ulm.de (G.B.); raphael.peter@uni-ulm.de (R.S.P.); dietrich.rothenbacher@uni-ulm.de (D.R.); 4 Division for Biochemistry of Joint and Connective Tissue Diseases, Department of Orthopedics, Ulm University, 89081 Ulm, Germany; rolf.brenner@uni-ulm.de; 5 Technical University of Dresden, University Center of Orthopedic and Trauma Surgery, 01069 Dresden, Germany; klaus-peter.guenther@uniklinikum-dresden.de; 6 Division of Clinical Epidemiology and Aging Research, German Cancer Research Center (DKFZ), 69120 Heidelberg, Germany; h.brenner@Dkfz-Heidelberg.de; 7 Division of Preventive Oncology, German Cancer Research Center (DKFZ) and National Center for Tumor Diseases (NCT), 69120 Heidelberg, Germany; 8 German Cancer Consortium (DKTK), German Cancer Research Center (DKFZ), 69120 Heidelberg, Germany; 9 Deutsches Herzzentrum München, Technische Universität München, 80636 Munich, Germany; 10 German Centre for Cardiovascular Research (DZHK), partner site Munich Heart Alliance, 80333 Munich, Germany

**Keywords:** growth differentiation factor-15, total mortality, osteoarthritis, prognosis

## Abstract

Background: Subjects with osteoarthritis (OA) are at increased risk for cardiovascular (CV) and all-cause mortality. Whether biomarkers improve outcome prediction in these patients remains to be elucidated. We investigated the association between growth differentiation factor 15 (GDF-15), a novel stress-responsive cytokine, and long-term all-cause mortality among OA patients. Methods: Within the Ulm Osteoarthritis Study, GDF-15 has been measured in the serum of 636 subjects, who underwent hip or knee arthroplasty between 1995 and 1996 (median age 65 years). Results: During a median follow-up of 19.7 years, a total of 402 deaths occurred. GDF-15 was inversely associated with walking distance. Compared to the bottom quartile (Q), subjects within the top quartile of GDF-15 demonstrated a 2.69-fold increased risk of dying (hazard ratio (HR) (95% confidence interval (CI)) 2.69 (1.82–3.96) adjusted for age, sex, BMI, smoking status, localization of OA, diabetes, maximum walking distance, total cholesterol, and cystatin C. Further adjustment for NT-proBNP, troponin I, and hs-C-reactive protein did not change the results appreciably (HR (95%CI) 1.56 (1.07–2.28); 1.75 (1.21–2.55); 2.32 (1.55–3.47) for Q2, Q3, and Q4 respectively, *p* for trend < 0.001). Conclusions: In subjects with OA, GDF-15 represents a potent predictor of decreased survival over >20 years, independently of conventional CV risk factors, renal, cardiac, and inflammatory biomarkers as well as walking disability, previously associated with increased mortality and lower extremity OA.

## 1. Introduction

Osteoarthritis (OA) is a very common disorder and patients have an excess risk, especially for cardiovascular death [1]. This excess burden of cardiovascular disease (CVD) in patients with chronic connective tissue disorders [2,3] could not be solely explained by overrepresentation of conventional cardiovascular (CV) risk factors in this high-risk population. A highly active pro-inflammatory milieu with altered immune response might further predispose them to accelerated atherogenesis [3]. Thus, adequate risk assessment for future CVD events in these subjects is essential not only for estimating prognosis, but also for individualized patient care. 

Although first attempts in this direction have already been undertaken, CVD risk prediction in these subjects is still challenging. Currently used risk stratification tools [4,5,6,7,8] regrettably are still not precise enough, leading to under- or overestimation of CVD risk in this patient population [9,10]. Even accounting for the pathophysiologic processes relevant for the underlying disease such as level of clinical disease activity (e.g., seropositivity for specific antibody and/or increased routine inflammatory parameters), extent of functional disability (e.g., joint erosions on X-ray), or disease duration and treatment modalities [11,12] did not lead to the desirable predictive accuracy for future CVD events [13]. More importantly, all these scores have been mainly applied in patients with rheumatic disorders, whereas data for other patient populations, such as with common age-related degenerative disease of the musculoskeletal system like OA, are still limited. 

Another way to improve risk stratification beyond risk factors (traditional and non-traditional) is by using novel circulating biomarkers. Indeed, several studies have already related numerous proteins (inflammatory or cardiac) to adverse CVD outcome in patients with chronic degenerative/rheumatic disorders [14,15]. However, their incremental value beyond traditional CV risk factors or even other protein biomarkers has been evaluated to a lesser extent.

Recently, growth differentiation factor-15 (GDF-15), a member of the transforming growth factor-beta superfamily [16], has been identified as a novel and promising candidate across a spectrum of CVD ranging from acute ischemic events through chronic atherosclerotic disease to manifest heart failure [17,18]. Moreover, elevated GDF-15 has been recognized as a powerful predictor of bleeding in subjects receiving dual antiplatelet therapy or in those on anticoagulant therapy [19,20]. In contrast to other established CVD biomarkers, GDF-15 is also strongly related to numerous non-cardiovascular conditions such as kidney diseases, diabetes mellitus, neoplasia and cancer-induced anorexia, or rheumatoid arthritis [17,18]. Even in young subjects without overt CVD, GDF-15 might represent an early indicator of CV risk development [21]. Finally, its strong association with all-cause mortality [22,23,24,25,26,27,28,29,30,31] might also imply a crucial role of GDF-15 in biological processes associated with ageing. However, data from patient populations with osteoarthritis are rare yet.

Considering the involvement of GDF-15 in controlling various physiological and pathological conditions, we thought to investigate whether GDF-15 is able to predict increased long-term risk of all-cause mortality beyond traditional risk factors and biochemical risk markers in a high-risk population for CVD, like patients with OA. 

## 2. Materials and Methods

### 2.1. Study Design and Population

The Ulm Osteoarthritis Study represents a multicenter prospective cohort study of OA patients with unilateral total hip or knee replacement between January 1995 and December 1996. Details of the study design have been reported elsewhere [32]. Briefly, at baseline, 809 consecutive patients, who underwent unilateral total hip or knee arthroplasty due to advanced OA, were eligible for the study. Exclusion criteria were age > 75 years, malignancies, inflammatory diseases, corticosteroid medication, or previous hip or knee joint replacement. Participation was voluntary and written informed consent was obtained from each subject upon entry into the study. The study protocol and study documents were approved by the Ethics Committee of Ulm University (No. 164/14) and were performed according to the principles of Good Clinical Practice and the Declaration of Helsinki.

For the present report, 173 participants with missing information on biomarker concentrations at baseline were excluded. In total, 636 subjects with all information on variables of interest were considered for the analyses. All-cause mortality was used as the primary outcome of the study. Death was ascertained by obtaining the survival status via the respective residents’ registration office. Mortality was assessed during follow-up (FU) at 6, 12, and 60 months after joint replacement and again, in the years 2014, 2015, and 2019 (last update was 11 June 2019). Information on living status could be traced for 98.4% of participants.

### 2.2. Data Collection 

All subjects underwent a baseline examination in a standardized manner according to the study protocol in Ulm, Augsburg, or Stuttgart (three cities in the South of Germany). Data were collected by standardized questionnaires in personal interviews and included demographic and lifestyle-related data (e.g., age, sex, weight, height, smoking status) and information on self-reported medical history (e.g., diabetes mellitus, hypertension, myocardial infarction, and heart failure). Finally, OA-related variables such as an assessment of maximum walking distance (in six categories) as well as the functionality subscale of the Western Ontario and McMaster University Osteoarthritis Index (WOMAC) (score range 0–68) used to specifically describe function in the operated joint at baseline assessment (preoperative) and the pain subscale of the WOMAC (pain score 0–20) as a measure of paint intensity were also applied.

### 2.3. Laboratory Methods 

Venous blood was drawn preoperatively under standardized conditions from all study participants after an overnight fasting period. All samples were stored at −80 °C until further analysis. No samples were inadvertently thawed during storage. All routine laboratory parameters were determined on the day of sampling by standard methods in the central laboratory of participating centers. 

The following biomarkers were measured in frozen serum samples in 2018: serum cystatin C was measured by immunonephelometry on a BNA II (Behring Co. Marburg, Germany) with an inter-assay coefficient of variation (CV) between 1.7% and 2.7%. High sensitivity C-reactive protein (hs-CRP) was measured using latex-enhanced nephelometry (NA-latex CRP, Behring Co., Marburg, Germany), calibrated with the WHO reference standard 85/506 (inter-assay CVs between 2.1% and 3.2%). N-terminal pro-B-type natriuretic peptide (NT-proBNP) concentrations were determined on an Elecsys (Roche Diagnostics, Penzberg, Germany) with a limit of detection (LoD) of 5.0 ng/L and an inter-assay CV < 5%. Concentrations of hs-cardiac troponin I (hs-cTnI) were measured on an Architect STAT (Abbott Diagnostics, Wiesbaden, Germany) with a LoD of 2.0 ng/L and inter-assay CVs between 3.8%, 3.9%, and 5.6% at three different concentrations. 

Baseline sCOMP was analyzed using a commercial sandwich ELISA against human COMP (BioVendor, Heidelberg, Germany; LOD < 0.4 ng/mL). The assay was run according to the manufacturer’s instructions, using a sample dilution of 1:50. Both inter- (CV 15.6%) and intra-assay variations (CV 2.8%) were assessed as quality control. The absorbance was detected at 450 nm and a reference wavelength at 630 nm by using the multimode microplate reader Infinite M200 Pro (Tecan Austria GmbH, Groedig, Austria).

Finally, GDF-15 serum concentration was measured by Electrochemiluminescence Immunoassay (ECLIA, Cobas Elecsys 411, Roche Diagnostics, Penzberg, Germany) with a measuring range of 27.6–12,700 ng/L, a LoD of 10 ng/L, and inter-assay CVs of 4.6% and 4.9% at two different concentrations. All analyses were run in a blinded fashion.

### 2.4. Statistical Analysis

Baseline demographic, clinical, and laboratory characteristics were compared in a descriptive way. For the present analysis, GDF-15 concentrations were categorized into quartiles (Q) with a cut-point of <780, 780−<1006, 1006–1279, and >1279 ng/L, respectively. Continuous variables were reported as medians, with their interquartile ranges (IQR, 25th and 75th percentile); categorical variables were presented as absolute frequencies (percentage and number). Measured biomarkers were natural log-transformed prior to the analysis, where appropriate. 

Partial Spearman correlation coefficients, adjusted for age and sex, were calculated to describe the correlation between GDF-15 and numerous variables of interest. 

Multivariable Cox proportional hazard analysis with various degrees of adjustment was carried out to investigate the predictive value of GDF-15 on long-term all-cause mortality. Concentrations of GDF-15 were divided into quartiles of their distribution (as outlined above), with quartile one as the reference quartile, or were used continuously (per unit increase after log-transformation). A basic model was adjusted for age (in years) and sex (male, female). A second model further included body mass index (BMI) (continuous kg/m^2^), smoking status (never, former, current), localization of the OA (hip, knee), history of diabetes mellitus (yes/no), serum total cholesterol (continuous), maximum walking distance (in categories), and continuous log-transformed cystatin C concentrations. The final model was additionally adjusted for continuous log-transformed concentrations of hs-CRP, hs-cTnI, and NT-proBNP. Results are reported as Hazard Ratios (HR) with their 95% confidence intervals (CIs). In addition, a dose–response relationship between GDF-15 and outcome was plotted with restricted cubic splines using four knots and the median concentration in the lowest category as the reference. The Cox proportional regression analysis was conducted for the whole follow-up period, as well as repeated under the same conditions using a differential FU time (i.e., after 5, 10, 20, and 24 years of FU). 

Finally, to investigate whether GDF-15 might improve risk assessment beyond the established risk predictors in OA patients, several measures of model accuracy were performed. First, to assess discrimination of events, areas under the receiver-operating characteristic curve (AUC) were calculated for models without (“basic” model) and with biomarkers (adding each of the biomarkers individually: hs-CRP, hs-cTnI, NT-proBNP, and GDF-15). The first, “basic” model contained age, sex, BMI, smoking status, localization of OA, diabetes mellitus, cholesterol, and log-transformed concentration of cystatin C. A second, “full” model contained in addition to the above mentioned variables and in addition hs-CRP, hs-cTnI, and NT-proBNP as reference models and then, GDF-15 was added. Furthermore, the net reclassification improvement (NRI) in cases and non-cases calculated by the Kaplan–Meier estimator was used to examine the predictive value of studied biomarkers according to the 1%, 5%, and 10% risk strata of the predicted probability for death after ten years [33]. The bootstrap method was used to create 95% CI for the NRI estimates [34]. Statistical analysis was performed using SAS version 9.4 (SAS Institute Inc., Cary, NC, USA) and R version 3.5.1 (The R Foundation for Statistical Computing, Vienna, Austria).

## 3. Results

In the total population of 636 participants with OA, the median (IQR) GDF-15 concentration at baseline was 1005.5 ng/L (780.0–1280.5 ng/L). Baseline demographic, clinical, and laboratory characteristics of the study participants in quartiles of the GDF-15 distribution are summarized in Table 1.

Subjects with higher GDF-15 concentrations were older and more likely to be females. Furthermore, the prevalence of diabetes mellitus and hypertension increased markedly with increasing GDF-15 concentrations. In addition, BMI, frequency of previous myocardial infarction, and percentage of never smokers tended to be higher in higher GDF-15 quartiles. No clear association was revealed throughout the GDF-15 quartiles with the presence of former or current smoking. Interestingly, the proportion of subjects who underwent total knee replacement was higher in the upper GDF-15 quartile concentrations compared to patients with hip OA. It should be noted here, however, that subjects with knee OA tended to be older compared to subjects with hip OA (median (IQR: 67 (64–71) years versus 62 (55–69) years). 

With regard to the laboratory parameters, subjects with higher GDF-15 concentrations had also higher concentrations of most investigated analytes (Table 1), with the only exception being for triglycerides. 

In addition, an inverse relationship has been observed between maximum walking distance and GDF-15 concentrations, with the higher values of GDF-15 associated with shorter walking distance. 

Next, we calculated age- and sex-adjusted partial Spearmen rank correlations coefficients between GDF-15 and cardiac, lipid, and renal parameters (Table 2). 

The strongest correlation was found for GDF-15 and cystatin C with a Rho of 0.371 (*p* < 0.001), followed by both troponins (Rho = 0.230; *p* < 0.001) and hs-CRP (Rho = 0.173; *p* < 0.001). Surprisingly, NT-proBNP and sCOMP were only modestly correlated to GDF-15 (Rho = 0.127 and Rho = 0.078, respectively) (Table 2).

Finally, we were interested in the prognostic evaluation of GDF-15 for all-cause mortality. The association between concentrations of GDF-15 at baseline and all-cause mortality over a median FU of 19.7 years was assessed both in quartiles of the GDF-15 distribution as well as continuously (per log unit (U) increase in GDF-15 concentration) (Table 3). 

During FU, a total of 402 (63.2%) deaths occurred. Deceased subjects demonstrated markedly higher median GDF-15 concentrations at baseline than non-cases (1120.5 vs. 796.7 ng/L, respectively, data not shown). Total mortality rate was found to be 36.4 deaths per 1000 person-years and increased from 14.7 per 1000 person-years in quartile 1 to a mortality rate of 61.7 deaths per 1000 person-years in the quartile 4 (Table 3).

The results of multivariable Cox proportional hazard analyses are presented in Table 3. After adjustment for age and sex, subjects within Q2, Q3, and Q4 of the GDF-15 distribution demonstrated a 2- to 3-fold increased risk of dying from all-causes, compared to those within the bottom quartile (Q1) (HR (95% CI) 1.85 (95% CI 1.30–2.63), 2.06 (95% CI,1.45–2.93), and 2.93 (95% CI, 2.05–4.18), respectively; *p* for trend <0.001). Further adjustment for well-established confounders such as BMI, smoking status, localization of OA, diabetes, maximum walking distance, total cholesterol, and cystatin C in model 2 led to only minimal reduction in estimates, with a HR of 1.64 (95% CI 1.12–2.38) for Q2 and a HR of 1.84 (95% CI 1.27–2.66) for Q3, and a HR of 2.69 (95% CI 1.82–3.96) for Q4, respectively. Most importantly, additional simultaneous adjustment for NT-proBNP, hs-cTnI, and hs-CRP did not change the results appreciably (HR (95%CI) 1.56 (95%CI 1.07–2.28), 1.75 (95%CI 1.21–2.55) and 2.32 (95%CI 1.55–3.47) for Q2, Q3, and Q4 versus Q1 respectively, *p* for trend <0.0001). Almost identical results in the different models were seen if GDF-15 concentrations were taken in the analysis as a continuous variable with a HR of 2.12 (95% CI 1.56–2.88) in the fully adjusted model. 

**Figure 1:**
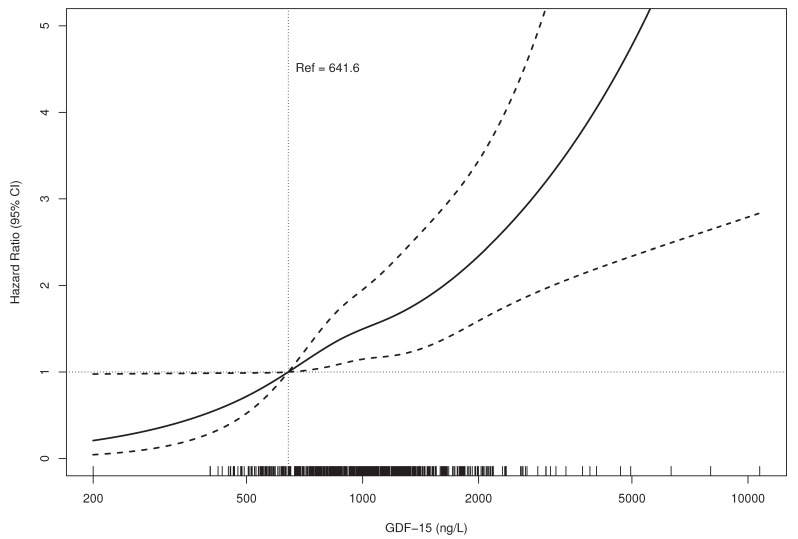
Relationship between GDF-15 Concentration and Mortality.(HR bold line, 95% CI dotted lines).Adjusted for age, sex, BMI, smoking status, localization of OA, diabetes, maximum walking distance, total cholesterol, and log-transformed concentrations of cystatin C, hs-CRP, hs-cTnI, and NT-proBNP (Model 3 from Cox proportional hazards analysis). BMI—body mass index; OA, osteoarthritis; hs-CRP—high-sensitive C-reactive protein; hs-cTnI—high-sensitive troponin I; NT-proBNP—N-terminal pro-B-type natriuretic peptide; GDF-15—growth differentiation factor-15.

Proportional hazard estimates for all-cause mortality across all ranges of GDF-15 concentrations based on a fully adjusted multivariable model (Model 3 in Table 3) are graphically presented in Figure 1. 

In addition, we repeated the Cox proportional analysis under the same levels of adjustments, truncating follow-up at 5-, 10-, and 20-years and saw no substantial changes in HRs with increasing FU period, documenting the robustness of the GDF-15 risk estimates over follow-up time (Figure S1 Supplemental Materials).

To investigate whether GDF-15 might improve risk assessment beyond established risk predictors, several measures of model accuracy were calculated, as shown in Table 4. 

The addition of log GDF-15 or other investigated biomarkers to the basic model led to only minimal improvement in the AUC, thereby indicating an almost negligible incremental value of these parameters for prediction of all-cause mortality. However, increase from 0.73 (95% CI, 0.71–0.76) to 0.76 (95% CI, 0.73–0.78) was seen when all biomarkers were considered together. Reclassification analysis in the deceased further showed only as slightly different from zero NRI (event NRI), whereas non-event NRI for GDF-15 beyond factors included in the basic model was found to be 0.06 (95%CI 0.00–0.13), indicating a correct downward reclassification of non-events. Interestingly, further reclassification (downwards) was seen if GDF-15 was evaluated additionally in the second, “full” model including other biomarkers (NRI_ne_ 0.04 (95%CI −0.01–0.09)); however, the 95% CI included the null-effect value.

## 4. Discussion

To the best of our knowledge, the present analysis represents the first prospective study investigating the prognostic value of the novel promising biomarker GDF-15 for all-cause mortality in subjects with OA considering a large number of established and new cardiovascular biomarkers. Our major finding showed higher GDF-15 concentration to predict worse survival both short- and long-term over >20 years in this high-risk population. Importantly, the observed association between increased GDF-15 concentrations and a higher risk of dying from all causes was independent not only of well-known potential confounders like traditional CVD risk factors or markers of renal dysfunction. Even after simultaneous adjustment for walking disability as well as for established strong predictors of future CVD events like hs-CRP, hs-TnI, and NT-proBNP, GDF-15 remained a potent risk indicator of all-cause death with a 2-times-higher rate of those in the top compared to the lowest GDF-15 quartile. Thus, in subjects with OA, GDF-15 provides additional prognostic information on all-cause mortality, which is not captured by conventional risk factors, “maximum walking distance” as a measure of gait disability, or other well-established biomarkers and may play a role in early risk stratification measures.

The independent association between elevated GDF-15 concentrations and short- or long-term total mortality has been already conclusively demonstrated among subjects from the general population as well as in various patient cohorts [17,18,22,23,24,25,26,27,28,29,30,31] and some of the studies had used the same analysis platform than our study [25,26,29]. Interestingly, several studies [23,30,31] have demonstrated that GDF-15 predicted all-cause mortality independently of and more accurately than other biomarkers like NT-proBNP, hs-CRP, or hs-cTn. Furthermore, in the general population [23,24], GDF-15 was the only biomarker additionally predictive for non-CVD and cancer mortality. Robust relations of GDF-15 to non-cardiovascular events, which was not seen for NT-proBNP, troponins, and hs-CRP, distinguishes it from other abovementioned CVD risk markers and suggests additional regulatory mechanisms for GDF-15 expression.

Indeed, GDF-15 represents a stress-responsive cytokine with multiple mechanisms of action [16]. While weakly expressed under physiological conditions, GDF-15 is highly secreted as a stress response to inflammation, oxidative stress, hypoxia, telomere erosion, and oncogene activation. GDF-15 has also been considered as a marker of frailty due to its association with mitochondrial dysfunction [35]. Nonetheless, biological processes that could explain a link between increased GDF-15 concentrations and various cardiac and non-cardiac outcomes are still not entirely elucidated. One might speculate that GDF-15 could play a dual (deleterious and protective) role in a variety of settings, as it e.g., has been recently proposed for cancer [16]. Premature or biological immunosenescence with accompanying “inflammaging” [36] might represent other possible mechanisms, relating GDF-15 to multiple age-related phenotypes and pathologies like OA, cancer, neurodegenerative disorders, frailty, or CVD. Two recently published proteome-based analyses [37,38] provided the first evidence on such associations. In the first study by Tanaka et al. [37], GDF-15 was found to be one of the strongest biomarkers associated with chronological age among 1301 tested proteins. Within the other analysis [38], GDF-15 has been demonstrated as a robust marker of senescence-associated secretory phenotypes, being simultaneously identified as a protein, highly secreted following all senescence-inducing stimuli, and as a biomarker, being highly related to aging.

Another unexpected finding from the present analysis was related to the fact that among OA subjects, GDF-15 was independently predictive for total mortality already at concentrations between 780 and 1006 ng/L (i.e., at Q2 of GDF-15 distribution with a median age of 65.0 years), where a HR of 1.56 (95% CI 1.07–2.28) was observed after adjustment for potential confounders, including other risk biomarkers. Several studies [26,28] have proposed a cut-off >1800 ng/L to identify high-risk subjects based on elevated concentrations of GDF-15. Although not directly comparable with the current analysis, data from the general population [23] or from several patient cohorts [26] demonstrated any meaningful association with all-cause mortality at much higher concentrations. Interestingly, the 50th percentile of GDF-15 concentration within the general population was found to be 945 ng/L at an age of 65 years [39]. Transferring these reference values to our results, one might conclude that subjects with OA have a 56% increased risk of dying, even being within the age-related reference limit of GDF-15.

Our study has several strengths that need to be mentioned. First, the present analysis was based on a well-characterized patient cohort including more than 600 subjects. Simultaneous assessment of novel and established biomarkers of different key pathways as well as a long FU period represent further strengths. Despite the intriguing results, several limitations of the current study also merit consideration. One of them is generalizability, since only Central Europeans aged 28 to 75 years were investigated. Furthermore, only subjects with advanced hip or knee OA, which required unilateral joint replacement, were included, which represent a selective population. No detailed information on long-term stability of the measured biomarkers in frozen samples was available, so we could not completely exclude that longer FU might influence the association between the risk marker and the disease outcome. However, in that case, we would expect rather a weakening of the associations found. Moreover, in another study, evaluating the 8-year stability of e.g., NT-proBNP serum samples stored at −80 °C with a maximum of one defrost cycle, we found an estimated recovery between 89.5% and 103% [40]. In addition, the platform used to measure GDF-15 in each study has to be considered. Finally, since all-cause mortality was the sole outcome in the present study, a predictive value of GDF-15 on CVD and non-CVD mortality has not been examined.

This is the first prospective study in patients with OA, demonstrating a strong prognostic value of elevated GDF-15 on decreased survival over >20 years. More importantly, the observed association was not affected by conventional CV risk factors as well as numerous well-established renal, cardiac, and inflammatory biomarkers. Although the results should preferably be verified in other cohorts, our findings suggest GDF-15 as a robust risk marker with a predictive value persisting even decades later. Thus, we believe that GDF-15, being not only strongly involved in biological processes associated with ageing but rather indicating an overall response to various stressors, might be a useful biomarker for future risk stratification or targeting preventive measures in such high-risk populations like subjects with OA.

## Figures and Tables

**Table 1:** Demographic, clinical, and laboratory characteristics of the study participants at baseline by quartiles of GDF-15.

		Q1 <780.0 ng/L	Q2 780.0–<1006.0 ng/L	Q3 1006.0–1279.0 ng/L	Q4 >1279.0 ng/L	Total
*n*		158	160	159	159	636
Age, years		58.0 (53.0/64.0)	65.0 (60.0/69.0)	67.0 (61.0/71.0)	70.0 (65.0/72.0)	65.0 (58.0/70.0)
Sex, % (*n*)	Male	46.8 (74)	32.5 (52)	35.8 (57)	27.7 (44)	35.7 (227)
	Female	53.2 (84)	67.5 (108)	64.2 (102)	72.3 (115)	64.3 (409)
Body Mass Index, kg/m²		27.3 (25.0/29.8)	27.8 (25.3/30.1)	27.8 (24.8/30.9)	28.7 (25.9/32.4)	27.8 (25.5/30.9)
Localization of OA, % (*n*)	Hip	63.9 (101)	56.3 (90)	51.6 (82)	38.4 (61)	52.5 (334)
	Knee	36.1 (57)	43.8 (70)	48.4 (77)	61.6 (98)	47.5 (302)
Smoking status, % (*n*)	Never	55.7 (88)	58.8 (94)	59.1 (94)	59.1 (94)	58.2 (370)
	Former	33.5 (53)	28.8 (46)	27.7 (44)	30.8 (49)	30.2 (192)
	Current	10.8 (17)	12.5 (20)	13.2 (21)	10.1 (16)	11.6 (74)
History of diabetes mellitus, % (*n*)		3.2 (5)	6.3 (10)	11.9 (19)	13.8 (22)	8.8 (56)
History of hypertension, % (*n*)		41.1 (65)	45.6 (73)	51.6 (82)	67.3 (107)	51.4 (327)
History of myocardial infarction, % (*n*)		3.8 (6)	1.3 (2)	4.4 (7)	5.7 (9)	3.8 (24)
History of heart failure, % (*n*)		6.3 (10)	16.9 (27)	17.0 (27)	33.3 (53)	18.4 (117)
Total cholesterol, mmol/L		5.6 (5.2/6.3)	5.7 (5.0/6.4)	5.8 (5.0/6.3)	5.8 (5.1/6.4)	5.7 (5.1/6.4)
Triglyceride, mmol/L		1.4 (1.0/2.0)	1.4 (1.0/2.1)	1.6 (1.0/2.3)	1.5 (1.1/2.2)	1.5 (1.0/2.1)
Cystatin C, mg/L		0.8 (0.7/0.9)	0.8 (0.8/0.9)	0.9 (0.8/1.0)	1.0 (0.9/1.2)	0.9 (0.8/1.0)
hs-CRP, mg/L		2.0 (1.0/3.8)	2.5 (1.1/4.9)	2.5 (1.4/6.2)	3.0 (1.8/6.3)	2.5 (1.3/5.0)
hs-cTnI, ng/L		3.1 (2.3/4.7)	3.7 (2.7/5.3)	3.9 (2.9/5.5)	5.3 (3.8/7.6)	3.9 (2.8/5.7)
NT-proBNP, ng/L		67.7 (32.7/118.3)	93.5 (49.9/167.5)	100.1 (62.0/162.3)	137.8 (84.6/269.1)	99.1 (52.2/83.9)
sCOMP, ng/mL		751.0 (545.3/980.8)	783.2 (586.8/1064.8)	826.4 (626.6/1138.6)	831.4 (626.2/1080.6)	797.1 (598.6/1044.5)
WOMAC pain score		12.0 (9.0/14.0)	11.5 (9.0/13.0)	12.0 (10.0/15.0)	13.0 (10.0/15.0)	12.0 (10.0/14.0)
WOMAC function score		39.5 (31.0/46.0)	39.5 (32.0/46.0)	39.0 (31.0/46.0)	40.0 (33.0/48.0)	39.5 (32.0/46.0)
Maximum walking distance	>1000 m, but limited	31.8 * (55)	24.9 * (43)	21.4 * (37)	22.0 * (38)	27.2 (173)
~1000 m or ~15 min		30.9 * (29)	19.2 * (18)	25.5 * (24)	24.5 * (23)	14.8 (94)
500–900 m or ~8–15 min		26.2 * (27)	27.2 * (28)	22.3 * (23)	24.3 * (25)	16.2 (103)
	300–500 m	18.0 * (15)	25.3 * (21)	26.5 * (22)	30.1 * (25)	13.1 (83)
	100–300 m	19.6 * (22)	27.7 * (31)	32.1 * (36)	20.5 * (23)	17.6 (112)
	<100 m	14.1 * (10)	26.8 * (19)	23.9 * (17)	35.2 * (25)	11.2 (71)

Data are expressed as a median (25th/75th percentile) or in column % (*n*) if not indicated otherwise (*) as row %. GDF-15—growth differentiation factor-15; Q—quartile; OA—osteoarthritis; hs-CRP—high-sensitive C-reactive protein; hs-cTnI—high-sensitive troponin I; NT-proBNP—N-terminal pro-B-type natriuretic peptide; sCOMP—serum cartilage oligomeric matrix protein; WOMAC—Western Ontario and McMaster University Osteoarthritis Index; m—meters; min—minutes.

**Table 2:** Correlation between GDF-15 and several biochemical parameters, adjusted for age and sex.

	Partial Spearman Rank Correlation Coefficients (Rho)	*p*-Values
Total cholesterol	−0.026	0.527
Triglyceride	0.093	0.036
Cystatin C	0.371	<0.001
hs-CRP	0.173	<0.001
hs-cTnI	0.230	<0.001
NT-proBNP	0.127	0.001
sCOMP	0.078	0.050

GDF-15—growth differentiation factor-15; Q—quantile; OA—osteoarthritis; hs-CRP—high-sensitive C-reactive protein; hs-cTnI—high-sensitive troponin I; NT-proBNP—N-terminal pro-B-type natriuretic peptide—sCOMP—serum cartilage oligomeric matrix protein.

**Table 3:** Association between GDF-15 concentration at baseline and risk of long-term all-cause mortality in subjects with osteoarthritis.

		Events/N	Rate per 1000 *p*-yr	Model 1HR (95% CI)	Model 2HR (95% CI)	Model 3HR (95% CI)
GDF-15	Quartile 1 (<780.0 ng/L)	48/158	14.7	1.00	1.00	1.00
	Quartile 2 (780.0–1006.0 ng/L)	100/160	35.0	1.85 (1.30–2.63)	1.64 (1.12–2.38)	1.56 (1.07–2.28)
	Quartile 3 (1006.0–1279.0 ng/L)	118/159	43.3	2.06 (1.45–2.93)	1.84 (1.27–2.66)	1.75 (1.21–2.55)
	Quartile 4 (>1279.0 ng/L)	136/159	61.7	2.93 (2.05–4.18)	2.69 (1.82–3.96)	2.32 (1.55–3.47)
	*p* for trend			<0.001	<0.001	<0.001
	Per log unit increase	402/636		2.41 (1.88–3.10)	2.34 (1.75–3.14)	2.12 (1.56–2.88)

Results from multivariable adjusted Cox proportional hazards model with all-cause mortality as dependent variable. Median (25th/75th percentile) follow-up time is 19.7 (12.6/22.9) years **Model 1** was adjusted for age and sex. **Model 2** was adjusted for age, sex, BMI, smoking status, localization of OA, diabetes, maximum walking distance, total cholesterol, and log-transformed concentration of cystatin C. **Model 3** was additionally adjusted for log-transformed concentrations hs-CRP, hs-cTnI, and NT-proBNP. N—number of observed subjects; *p*-yr—person-years; HR—hazard ratio; CI—confidence interval; GDF-15—growth differentiation factor-15; OA for osteoarthritis; hs-CRP—high-sensitive C-reactive protein; hs-cTnI—high-sensitive troponin I; NT-proBNP—N-terminal pro-B-type natriuretic peptide.

**Table 4:** Measures of model discrimination and reclassification for all-cause mortality in subjects with osteoarthritis.

	AUC (95% CI)	NRI_e_ (95% CI)	NRI_ne_ (95% CI)
Basic model ^a^	0.73 (0.71/0.76)		
Basic model + ln(hs-CRP)	0.73 (0.71/0.76)	−0.01 (−0.06/0.03)	0.01(−0.01/0.06)
Basic model + ln(hs-cTnI)	0.74 (0.72/0.77)	−0.01 (−0.08/0.06)	0.06 (0.02/0.14)
Basic model + ln(NT-proBNP)	0.74 (0.72/0.77)	0.05 (−0.05/0.09)	0.09 (0.01/0.17)
Basic model + ln(GDF-15)	0.74 (0.72/0.77)	0.02 (−0.06/0.10)	0.06 (0.00/0.13)
Full model ^b^	0.75 (0.72/0.77)		
Full model + ln(GDF-15)	0.76 (0.73/0.78)	−0.01 (−0.06/0.07)	0.04 (−0.01/0.09)

^a^ Basic model (Model 2 from Cox proportional hazards analysis) was adjusted for age, sex, BMI, smoking status, localization of OA, diabetes, maximum walking distance, cholesterol, and log-transformed concentration of cystatin C. ^b^ Full model (Model 3 from Cox proportional hazards analysis) was adjusted for age, sex, BMI, smoking status, localization of OA, diabetes, maximum walking distance, total cholesterol, and log-transformed concentrations of cystatin C, hs-CRP, hs-cTnI, and NT-proBNP. AUC—areas under the receiver-operating characteristic curve; NRI_e_—event net reclassification index; NRI_ne_—non-event net reclassification index; hs-CRP—high-sensitive C-reactive protein; hs-cTnI—high-sensitive troponin I; NT-proBNP—N-terminal pro-B-type natriuretic peptide; GDF-15—growth differentiation factor-15.

## Data Availability

Due to ethical restrictions regarding data protection issues and the study-specific consent text and procedure, the data cannot be made publicly available, but data are available to all interested researchers upon request.

## Supplementary Materials

The following are available online at https://www.mdpi.com/2077-0383/9/10/3107/s1, Figure S1: Cox Proportional Regression Analysis for Total Mortality Using Different Follow-up Periods.
